# Serum high-mobility group box 1 protein level correlates with the lowest SaO_2_ in patients with sleep apnea: a preliminary study

**DOI:** 10.1016/j.bjorl.2020.11.019

**Published:** 2021-01-02

**Authors:** Hyun Jin Min, Joon Soon Park, Kyung Soo Kim, Miran Kang, Ju Hee Seo, Joo-Heon Yoon, Chang-Hoon Kim, Hyung-Ju Cho

**Affiliations:** a Chung-Ang University College of Medicine, Department of Otorhinolaryngology-Head and Neck Surgery, Seoul, Republic of Korea; b Chung-Ang University Hospital, Biomedical Research Institute, Seoul, Republic of Korea; c Yonsei University College of Medicine, Department of Otorhinolaryngology, Seoul, Republic of Korea; d Yonsei University College of Medicine, The Airway Mucus Institute, Seoul, Republic of Korea

**Keywords:** HMGB1 protein, Hypoxia, Obstructive sleep apnea, Oxygen saturation (SaO_2_)

## Abstract

**Introduction:**

Serum level of high-mobility group box 1 protein is reportedly correlated with the severity of obstructive sleep apnea.

**Objective:**

We tried to evaluate the possibility of using the serum high-mobility group box 1 protein level as a biologic marker in obstructive sleep apnea patients.

**Methods:**

We generated a chronic intermittent hypoxia murine model that reflected human obstructive sleep apnea. Obstructive sleep apnea patients who underwent polysomnography were prospectively enrolled. Serum samples were obtained from mice and obstructive sleep apnea patients, and the serum high-mobility group box1 protein level was measured by enzyme-linked immunosorbent assay.

**Results:**

Serum high-mobility group box 1 protein level was 56.16 ± 30.33 ng/mL in chronic intermittent hypoxia and 18.63 ± 6.20 ng/mL in control mice (*p* < 0.05). The mean apnea-hypopnea index and respiratory disturbance index values of enrolled obstructive sleep apnea patients were 50.35 ± 27.96 and 51.56 ± 28.53, respectively, and the mean serum high-mobility group box 1 protein level was 30.13 ± 19.97 ng/mL. The apnea–hypopnea index and respiratory disturbance index were not significantly correlated with the serum high-mobility group box 1 protein level (*p* > 0.05). Instead, this protein level was significantly correlated with lowest arterial oxygen concentration (SaO_2_) (*p* < 0.05).

**Conclusion:**

High-mobility group box 1 protein may be involved in the pathogenesis of obstructive sleep apnea, and the possibility of this protein being a useful biologic marker in obstructive sleep apnea should be further evaluated.

## Introduction

Obstructive sleep apnea (OSA), which is characterized by recurrent episodes of breathing cessation or reduction during sleep, is the most common sleep-related breathing disorder.^1^ OSA induces shortage of the oxygen amount required during sleep and results in increased oxidative stress. Therefore, oxidative stress has been suggested as one of the underlying pathomechanisms that explain the relationship between OSA and OSA-related complications, such as hypertension, endothelial dysfunction, and ischemic heart disease.^2^ For example, OSA-induced intermittent hypoxia caused influx of free radicals and reactive oxygen species (ROS), and the increased level of ROS induced direct damage to the myocardium. The increased ROS during sleep also upregulated the adhesion molecules that can damage the endothelium.^3^ It was further demonstrated that the treatment of OSA decreased the oxidative stress, as proven by the decreased level of oxidative stress markers in the plasma.^4^ Although the molecular mechanisms leading to oxidative stress in OSA have not been properly elucidated, hypoxia and increased ROS levels are likely responsible for the oxidative damage in patients with OSA.^2^

High-mobility group box 1 protein (HMGB1) is a ubiquitous, abundant, and evolutionarily- conserved protein in eukaryotes that mediates DNA binding and functions as a non-histone DNA chaperone molecule in the nucleus. HMGB1 was rediscovered as a late product of endotoxin-stimulated macrophages that can be secreted into the extracellular area passively or actively through the cytoplasm.^5^ In the extracellular area, HMGB1 binds to receptors, such as toll-like receptor 2 (TLR2) and TLR4, and transfers inflammatory signals. Therefore, the mechanisms of HMGB1 translocation are considered important for controlling immunologic activity. Recently, oxidative stress has been found to be a critical factor in determining the cytokine function of HMGB1.^6^, ^7^ Hypoxia-induced ROS is reportedly important for the translocation of HMGB1 into the extracellular area.^8^ In addition, the redox status of HMGB1 determines its function in that the reduced and oxidized HMGB1 proteins have opposite functions.^7^ This finding of oxidative stress induced by hypoxic events being an important mechanism both in the pathogenesis of OSA and the function of HMGB1 led us to link HMGB1 with OSA.

A previous report demonstrated that the serum level of HMGB1 was higher in OSA patients than in controls, and the serum HMGB1 level was correlated with respiratory disturbance index (RDI). After continuous positive airway pressure (CPAP) treatment, the HMGB1 level returned to normal.^9^ However, there has been no further study that evaluated the serum HMGB1 level in OSA patients.

We hypothesized that HMGB1 can be used as a relevant parameter in the evaluation of OSA because oxidative stress is strongly associated with OSA.^10^, ^11^ In this study, we aimed to evaluate the relationship between HMGB1 levels and polysomnographic (PSG) findings and further support our hypothesis using a chronic intermittent hypoxia (CIH) murine model.

## Methods

### CIH murine model

The murine model of CIH was created according to a previously-described protocol (Supplementary Fig. 1).^12^ CIH is developed as a OSA animal model to investigate essential pathogenesis of OSA-related complications, because chronic exposure to hypoxia-reoxygenation is a major perturbation that has been shown to significantly and independently increase both the morbidities and mortality of patients with OSA.^13^ C57BL/6J adult male mice (8 weeks old) were randomly divided into two groups, each consisting of six mice, and were placed in identical chambers. The CIH group was exposed to 4 weeks of CIH (12 daylight hours per day), whereas the control group was maintained under normal oxygenation conditions. Mice were transferred to a customized CIH chamber that was connected to a gas control delivery system (Live Cell Instrument, Seoul, South Korea) during CIH. Each 120 s cycle included a stage where the O_2_ in the chamber was maintained at a nadir concentration of 5%, followed by restoration to 21%.

Mice were euthanized the day after the final hypoxic exposure, and blood samples were collected via cardiac puncture. Euthanizing mice before detecting serum HMGB1 levels has been commonly performed in previous studies and does not significantly affect the experimental results.^8^ Serums were centrifuged immediately, and serum samples were obtained and maintained at −70 °C until used for experimental procedures. HMGB1 levels were evaluated using the obtained serum samples.

### Patients

The protocol for human data collection was approved by the institutional review board of the Yonsei University College of Medicine (4-2018-0304, 4-2014-0246). Informed consent was obtained from all participants. Patients aged 19–70 years who visited the otorhinolaryngology department of Severance Hospital were enrolled in this study.

All participants were evaluated using PSG. All recordings were scored based on 30 s epochs according to the American Academy of Sleep Medicine (AASM) criteria.^14^ The sleep stages were scored using the standard criteria. Apnea was defined as a cessation of airflow for 10-s, and hypopnea was defined as a 30% reduction of airflow or respiratory movements accompanied by a 3% decrease in arterial blood oxygen saturation and/or followed by an arousal. The apnea-hypopnea index (AHI) was calculated for all patients, and only those with an AHI of >5 were enrolled. Patients with an AHI of 5–15 were assigned to the mild OSA group, those with an AHI of 16–30 to the moderate OSA group, and those with an AHI > 30 to the severe OSA group, following a previous study.^15^

Blood samples were collected in EDTA-containing tubes and anticoagulant-free tubes and were immediately centrifuged at 2500 g for 5-min. The separated serum samples were collected and stored at −80 °C until used for enzyme-linked immunosorbent assay (ELISA).

### Measurement of serum HMGB1 level

The serum levels of HMGB1 were measured using ELISA with the HMGB1 ELISA kit (Shino-Test Corp., Tokyo, Japan) following the manufacturer’s protocol. ELISA was performed following the “normal range” procedure, with sensitivity ranging from 0 to 80 ng/mL.

### Statistical analysis

Statistical analyses were performed using SPSS 23.0 (IBM Corp., Armonk, NY, USA). Data were shown as mean ± standard deviation. Spearman’s test was used for correlation analysis between continuous variables. Univariate and multivariate regression analyses were performed to determine the factors that significantly correlated with the HMGB1 level; *p*-values of 0.05 or less were considered statistically significant.

## Results

### CIH murine model

**Figure 1:**
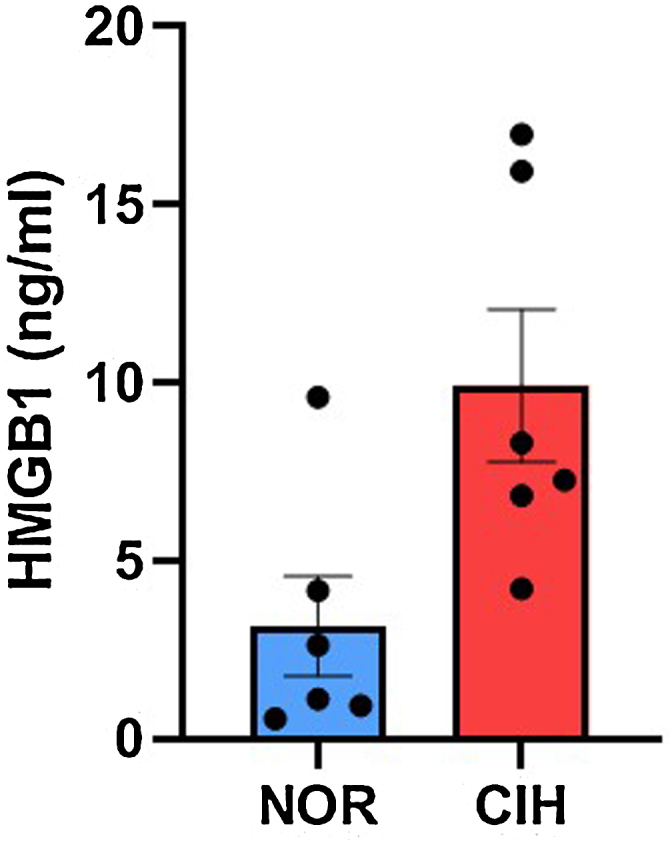
The serum level of HMGB1 increased in the CIH mice model, compared to the normal range of the control group (NOR). CIH, chronic intermittent hypoxia; NOR, normal range.

We generated a CIH murine model and control group (n = 6, in each). Serum samples were obtained from both groups and compared. The level of serum HMGB1 was significantly higher in the CIH group (9.91 ± 5.25 ng/mL) than in the control group (3.16 ± 3.42 ng/mL) (Fig. 1).

### Human subjects

**Figure 2:**
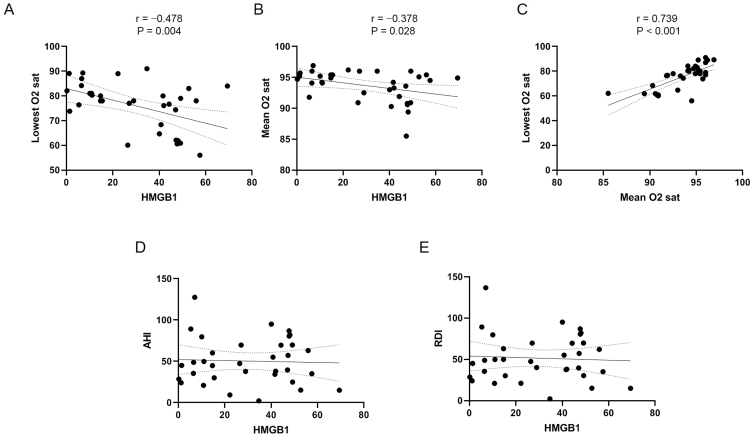
Association between HMGB1 levels and SaO_2_, apneahypopnea Index (AHI), and respiratory disturbance Index (RDI). (A) The lowest and (B) the mean SaO_2_ were negatively associated with the serum level of HMGB1. (C) The lowest and the mean SaO_2_ were associated with each other. (D) The AHI and (E) RDI were not associated with the serum level of HMGB1.

In the next step, we prospectively enrolled human OSA patients for whom PSG was performed. A total of 34 patients (32 men and 2 women) with OSA were enrolled in the study. The mean age was 41.85 ± 15.30 years. Eight patients (23.52%, 8/34) were diagnosed with hypertension, and four were diagnosed with diabetes (11.76%, 4/34). The mean serum HMGB1 level was 30.13 ± 19.97 ng/mL (Table 1). The mean AHI was 50.35 ± 27.96, and the mean RDI was 51.56 ± 28.53 (Table 2). The mean and lowest arterial oxygen concentrations (SaO_2_) were 93.66% ± 2.47% and 75.97% ± 9.70%, respectively. Most of the enrolled patients had severe OSA (26 of 34 patients), and two patients had mild OSA. The mean minimum SaO_2_ was 96.1% in the mild OSA group, 95.01% ± 0.59% in the moderate OSA group, and 73.26% ± 9.30% in the severe OSA group. Statistical analysis with the serum HMGB1 level and PSG results showed that the serum HMGB1 level was significantly correlated with the mean and lowest SaO_2_ (Fig. 2 A and B), and the mean SaO_2_ was also associated with the lowest SaO_2_ (Fig. 2C). However, the serum HMGB1 level was not correlated with AHI and RDI (Fig. 2D and E). In the univariate and multivariate regression analyses of the evaluated clinical and PSG factors, the lowest SaO_2_ was the only factor that remained significantly associated with the serum HMGB1 level (Table 3).

**Table 1:** Clinical characteristics of the enrolled subjects.

Parameter	Value
n	34
Sex, male/female	32/2
Age, years	41.85 ± 15.30
Body mass index, kg/m^2^	26.73 ± 3.48
Presence of hypertension	23.52% (8/34)
Presence of diabetes	11.76% (4/34)
Smoking history	
Current smoker	23.52% (8/34)
Former smoker	26.47% (9/34)
Non-smoker	50.0% (17/34)
High-mobility group box 1 (ng/mL)	30.13 ± 19.97

**Table 2:** Results of the sleep study on enrolled subjects.

Parameter	Value
AHI, times/h	50.35 ± 27.96
RDI, times/h	51.56 ± 28.53
AI, times/h	31.09 ± 25.06
Mean SaO_2_, %	93.66 ± 2.47
Lowest SaO_2_, %	75.97 ± 9.70
Sleep efficiency, %	86.58 ± 12.20
Longest apnea duration, s	64.11 ± 34.10
Longest hypopnea duration, s	55.39 ± 224.22
Mean apnea duration, s	28.80 ± 9.66
Mean hypopnea duration, s	27.42 ± 9.86
Mean ESS score	8.54 ± 4.69

AHI, apneahypopnea Index; AI, arousal index; ESS, epworth sleepiness scale; RDI, respiratory disturbance Index.

**Table 3:** Univariate and multivariate linear regression analyses to identify factors associated with the serum HMGB1 level.

Variables	Univariate		Multivariate	
	β (SE)	*p*-Value	β (SE)	*p*-Value
Age	−0.093 (0.230)	0.601	−0.140 (0.210)	0.389
Sex	−0.084 (14.773)	0.637	−0.100 (13.421)	0.537
BMI	0.0140 (1.024)	0.935	−0.100 (0.949)	0.544
AHI	−0.043 (0.126)	0.809		
RDI	−0.064 (0.125)	0.723		
AI	0.089 (0.144)	0.089		
Mean SaO_2_	−0.378 (1.359)	0.028		
Lowest SaO_2_	−0.478 (0.322)	0.004	−0.512 (0.340)	0.004
Longest apnea duration	0.137 (0.103)	0.441		
ESS score	0.118 (0.799)	0.526		

AHI, apneahypopnea Index; AI, arousal index; BMI, body mass index; ESS, epworth sleepiness scale; RDI, respiratory disturbance Index; SE, standard error.

## Discussion

The changes in normal physiology due to metabolic and endocrine dysfunction, chronic inflammation, stress, and hypoxia in patients with OSA are associated with alterations in serum levels of various molecules.^16^ Among these changes, we focused on the hypoxemia events and HMGB1 levels. Hypoxic conditions increase the level of ROS and ROS-induced oxidative stress. Oxidative stress is an important mechanism in extracellular translocation of HMGB1, and a previous study proved the correlation of the serum HMGB1 level with RDI in OSA patients.^9^ The major finding of the present study is that the serum HMGB1 level, which is a DAMP and mediator of innate immunity, was elevated in the CIH murine model but was not correlated with the AHI and RDI. Instead, the lowest SaO_2_ level was significantly correlated with serum HMGB1 levels.

A series of inflammatory reactions is proved to be associated with OSA. For example, activation of monocytes^17^ and activation and proliferation of T lymphocytes^18^ have been demonstrated in OSA. Furthermore, peripheral B cells and natural killer T-cells (NKT) were reduced in patients with OSA.^19^ These immune cells and immune cell markers were associated with the AHI and the mean and lowest SaO_2_.^20^ HMGB1 is a ubiquitous protein that is present in various types of cells and can be released into the extracellular area after stimulation. In the extracellular area, HMGB1 functions as a DAMP, also known as alarmin, which signals cellular damage and activates the innate immune system.^21^ In this study, we found that the extracellular level of HMGB1 was significantly elevated in the serum of CIH mice, suggesting that the hypoxic events during sleep increased the extracellular HMGB1 level. This is in accordance with our previous findings that hypoxic conditions increased the ROS level and HMGB1 secretion into the extracellular area, suggesting that HMGB1 is a useful marker for estimating the level of oxidative stress.^8^ As extracellular HMGB1 can function as a DAMP, elevated serum level of HMGB1 may be associated with systemic inflammatory events during the pathogenesis of OSA. Therefore, further studies are needed to identify the linkage between OSA and extracellular HMGB1. Interestingly, it has been reported that the level of oxidative stress markers in the saliva was associated with the severity of OSA and that CPAP treatment significantly decreased the morning concentrations of these markers in the saliva.^2^ As blood sampling is invasive, monitoring the level of HMGB1 in saliva and its diurnal variation would be another approach in evaluating HMGB1 as a biomarker that reflects the level of oxidative stress in OSA.

To our knowledge, there has been only one previous study that evaluated the serum level of HMGB1 in patients with OSA.^9^ In that study, the serum level of HMGB1 positively correlated with the RDI, which is not supported by our findings. Their characteristics of the enrolled subjects regarding the number of subjects, gender composition, and mean age were similar to this study. This discrepancy means that a large population-based study should be performed to establish the serum HMGB1 level as an important biologic marker in OSA.

The novelty of our study is that we evaluated the serum levels of HMGB1 in both human subjects and a murine model. As CIH is regarded as the key path mechanism in OSA, CIH murine models are widely used for studying OSA. Our finding that CIH mice showed a higher level of serum HMGB1 supports the notion that extracellular HMGB1 in the serum may play a role in the pathogenesis of OSA. In this study, we followed our previous CIH model, which includes 4 weeks of CIH. There is a possibility that duration of CIH could affect HMGB1 level, and the difference of HMGB1 level in mouse model with longer durations of CIH requires further examination.

The present study has several limitations. First, it does not establish a causal relationship between HMGB1 and OSA. This limits our findings as a preliminary study, and further studies should be performed about how the serum level of HMGB1 can have clinical value as a biomarker. Second, this study is based on a small population and does not include normal human subjects. There is a possibility that age, gender, and the presence of underlying hypertension could differentially affect the level of serum HMGB1. It has been reported that serum levels of HMGB1 significantly decrease with age in healthy subjects.^22^ Furthermore, as most enrolled patients in this study had severe OSA, our findings may not be in accordance with mild OSA. Third, we did not evaluate the relationship between oxygen desaturation index (ODI) or percentage of total sleep time with oxygen desaturation and the serum HMGB1 level. Recent studies suggest that patients with a similar AHI may show different pathophysiology according to nocturnal hypoxia, such as total sleep time with oxygen saturation <90%90 % or ODI. Further studies are needed to evaluate the relationship between nocturnal hypoxia and HMGB1 level.^23^ Finally, we only evaluated the level of HMGB1 and did not evaluate other molecules representative of oxidative stress in patients with OSA.

## Conclusions

Although there was a previous study showing that the serum HMGB1 level reflects the severity of disease in OSA patients, we found that the serum level of HMGB1 was not correlated with the AHI and RDI. Instead, we found that serum HMGB1 was significantly elevated in CIH mice and that the serum HMGB1 level was significantly correlated with the lowest SaO_2_ level in OSA patients. Further studies with a larger population would help evaluate the usefulness of HMGB1 as a biological marker of OSA and its consequences by the elevated HMGB1.

## Author’s contribution

H.J. Min wrote the manuscript. J.S. Park, M. Kang, and J.H. Seo performed the *in vitro* and *in vivo* experiments. K.S. Kim evaluated the data of human subjects with OSA. J.H. Yoon and C.H. Kim reviewed the manuscript. H.J. Cho designed the experimental procedures and is a guarantor of the manuscript.

## Funding

This work was supported by a grant from the National Research Foundation of Korea (NRF) funded by the Korean government (NRF-2017R1A1A1A05000760 to H.J. Min) and partially supported by a research grant from the Biomedical Research Institute, Chung-Ang University Hospital (2018). This research was also supported by the Basic Science Research Program through the NRF of Korea funded by the Ministry of Education (NRF-2018R1D1A1A02049236 to H.J. Cho).

## Conflicts of interest

The authors declare no conflicts of interest.
