# Supplementary material for: Serum high-mobility group box 1 protein level correlates with the lowest SaO2 in patients with sleep apnea: a preliminary study
Source: Braz J Otorhinolaryngol. 2021 Jan 2;88(6):875–81. doi: 10.1016/j.bjorl.2020.11.019 (PMC9615530; doi:10.1016/j.bjorl.2020.11.019)
Supplement: Supplementary file 1 [file mmc1.docx]

**Supplementary figure legend**

**Fig. S1** A schematic of the setting of the chronic intermittent hypoxia murine model experiment
